# Efficient inhibition of tumor angiogenesis and growth by a synthetic peptide blocking S100A4-methionine aminopeptidase 2 interaction

**DOI:** 10.1038/mtm.2015.8

**Published:** 2015-04-01

**Authors:** Takahiro Ochiya, Keizo Takenaga, Masataka Asagiri, Kazumi Nakano, Hitoshi Satoh, Toshiki Watanabe, Shinobu Imajoh-Ohmi, Hideya Endo

**Affiliations:** 1 Division of Molecular and Cellular Medicine, National Cancer Center Research Institute, Tokyo, Japan; 2 Department of Life Science, Shimane University School of Medicine, Izumo, Japan; 3 The Institute of Medical Science, The University of Tokyo, Tokyo, Japan; 4 Department of Medical Genome Sciences, Laboratory of Tumor Cell Biology, Graduate School of Frontier Sciences, The University of Tokyo, Tokyo, Japan

## Abstract

The prometastatic calcium-binding protein, S100A4, is expressed in endothelial cells, and its downregulation markedly suppresses tumor angiogenesis in a xenograft cancer model. Given that endothelial S100A4 can be a molecular target for inhibiting tumor angiogenesis, we addressed here whether synthetic peptide capable of blocking S100A4-effector protein interaction could be a novel antiangiogenic agent. To examine this hypothesis, we focused on the S100A4-binding domain of methionine aminopeptidase 2, an effector protein, which plays a role in endothelial cell growth. Overexpression of the domain in mouse endothelial MSS31 cells reduced DNA synthesis, and the corresponding synthetic peptide (named NBD) indeed interacted with S100A4 and inhibited capillary formation *in vitro* and new blood vessel formation *in vivo.* Intriguingly, a single intra-tumor administration of the NBD peptide in human prostate cancer xenografts significantly reduced vascularity, resulting in tumor regression. Mechanistically, the NBD peptide enhanced assembly of nonmuscle myosin IIA filaments along with Ser1943 phosphorylation, stimulated formation of focal adhesions without phosphorylation of focal adhesion kinase, and provoked G1/S arrest of the cell cycle. Altogether, the NBD peptide is a potent inhibitor for tumor angiogenesis, and is the first example of an anticancer peptide drug developed on the basis of an endothelial S100A4-targeted strategy.

## Introduction

S100A4 is a member of the S100 family of EF hand calcium-binding proteins and has been well documented as a proinvasive and prometastatic protein (for review see refs. 1,2). In fact, many studies including ours have shown that downregulation of S100A4 by siRNA and antisense strategies reduce the invasion and metastatic potential of tumor cells.^1–4^ Studies on S100A4-interacting proteins have identified various intracellular proteins such as F-actin,^5^ tropomyosin,^6^ the heavy chain of nonmuscle myosin-IIA (NMIIA),^7,8^ Liprin-β1,^9^ the tumor suppressor p53,^10,11^ and MDM2.^12^ These effector proteins indicate an intracellular role for S100A4 in cytoskeletal dynamics, cell motility, cell adhesion, and apoptosis. In fact, S100A4 has been shown to be involved in assembly of NMIIA filaments,^13^ stimulation of cell motility,^14^ enhancement of certain members of metalloproteinase family,^15^ stimulation of epithelial-mesenchymal transition,^16^ and modulation of transcription of p53-responsive genes such as mdm2, Bax, p21, and thrombospondin-1.^17^ Nevertheless, the precise molecular mechanisms by which S100A4 exerts these functions remain unclear.

Methionine aminopeptidase 2 (MetAP2) has been reported to catalyze the cotranslational removal of the initiator methionine residue from nascent peptides in eukaryotes.^18^ However, recent studies have shown that MetAP2 from yeast, rat, and human does not have MetAP activity. Instead, it is cleaved by auto-proteolysis into the C-terminal p52 fragment (108–480 amino acid residues) that harbors the binding regions to eukaryotic initiation factor 2γ (eIF2γ) and extracellular signal-regulated kinase 1/2 (ERK1/2) and the N-terminal p26 fragment (1–107 amino acid residues) that has the POEP (protection of eIF2α phosphorylation) activity. Thus, MetAP2 plays important roles in the regulation of phosphorylation of eIF2α and ERK1/2 (for review see ref. 19). Knockdown of the expression of MetAP2 in various mammalian cells results in phosphorylation of eIF2α, leading to the decreased rate of global protein synthesis and cell growth.^19^ MetAP2 has also been reported as a physiologically relevant target for potent antiangiogenic agents including fumagillin, TNP-470 and their analogs.^20,21^ Fumagillin binds covalently to the H231 residue of MetAP2 and inhibits MetAP2’s auto-proteolytic activity, and decreases its turnover rate, resulting in the inhibition of the activity of ERK1/2 and cell growth.^19^ Knockdown of MetAP2 using small interfering RNA or homologous recombination suppresses the growth of endothelial cells.^20^ Therefore, MetAP2 is thought to play a role in angiogenesis,^20–22^ although a contradicting report exists.^23^

We previously reported that MetAP2 is an effector protein of S100A4; that is, an *in vitro* pull-down assay showed that S100A4 binds to MetAP2 in a calcium-dependent manner and that the binding site of S100A4 is located within 60 amino acid residues from 170 to 229 of MetAP2.^24^ This interaction suggested the involvement of intracellular S100A4 in endothelial cell function (*e.g.*, angiogenesis). However, although several reports have shown that S100A4 acts upon endothelial cells as an extracellulary-acting angiogenic factor,^25,26^ its intracellular role in regulating angiogenesis had remained totally unclear. To resolve this issue, we recently performed a siRNA-mediated S100A4 knockdown in endothelial cells and revealed a critical role of endothelial S100A4 in *in vitro* tube formation as well as in tumor angiogenesis in a xenograft cancer model,^27^ highlighting endothelial S100A4 as a molecular target for inhibiting tumor angiogenesis.

The structure of active S100A4 was revealed in a recent report that showed that, upon calcium binding, dimeric S100A4 undergoes a significant conformational change, which opens up a hydrophobic target-binding pocket that is capable of binding to effector proteins.^28^ We hypothesized that a blockade of the binding pocket by peptides would inhibit S100A4-effector protein interactions, thereby leading to the suppression of the S100A4 function. Because the discovery of modulators of protein-protein interactions is an emerging field of drug discovery, we investigated here the efficacy of S100A4-binding domain peptide of MetAP2 on S100A4 function. The results obtained demonstrate the peptide as a potent inhibitor of tumor angiogenesis causing tumor regression.

## Results

### Inhibition of DNA synthesis of endothelial cells by the S100A4 binding domain (SBD) of MetAP2

**Figure 1:**
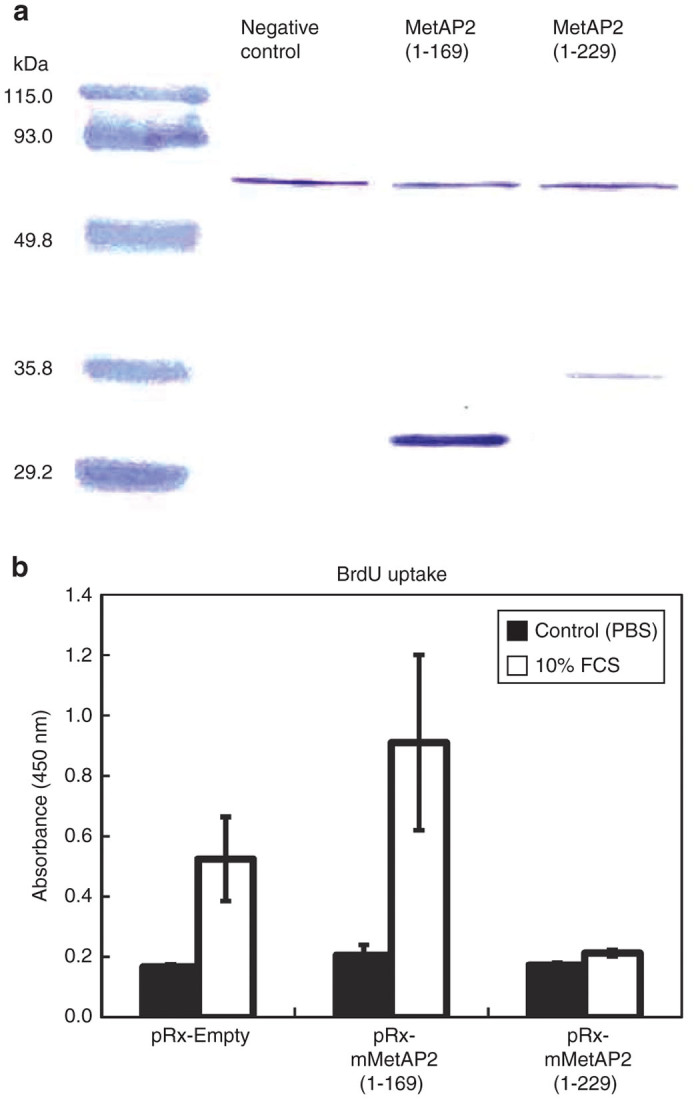
Suppression of DNA synthesis by overexpression of the SBD. (**a**) Recombinant protein expression in MSS31 cells retrovirally infected with the indicated vectors. MetAP2(1–169) and MetAP2(1–229) proteins were detected by western blot analysis using an anti-MetAP2 antibody. The 86 kD bands possibly derived from endogenous MetAP2 served as internal controls. (**b**) DNA synthesis in MSS31 cells retrovirally infected with the indicated vectors. DNA synthesis was determined by BrdU uptake using the Cell Proliferation ELISA BrdU kit. Black columns, untreated; open columns, 10% FBS-stimulated.

On the basis of our previous report that S100A4 physically interacts with MetAP2,^24^ we intended to block their interaction with the binding-domain, SBD, in endothelial cells. Since the SBD locates within the region between amino acid residues 170 and 229 of MetAP2, we first evaluated the effect of overexpression of a truncated form of MetAP2, MetAP2(1–229), along with its control, MetAP2(1–169), on DNA synthesis of MSS31 cells (Figure 1a). Infection of MSS31 cells with retroviruses expressing MetAP2(1–229), but not MetAP2(1–169), resulted in a marked suppression of serum-induced DNA synthesis (Figure 1b), suggesting that the SBD is capable of inhibiting endothelial cell growth.

### Binding of the SBD peptide to Ca^2+^-bound S100A4

**Figure 2:**
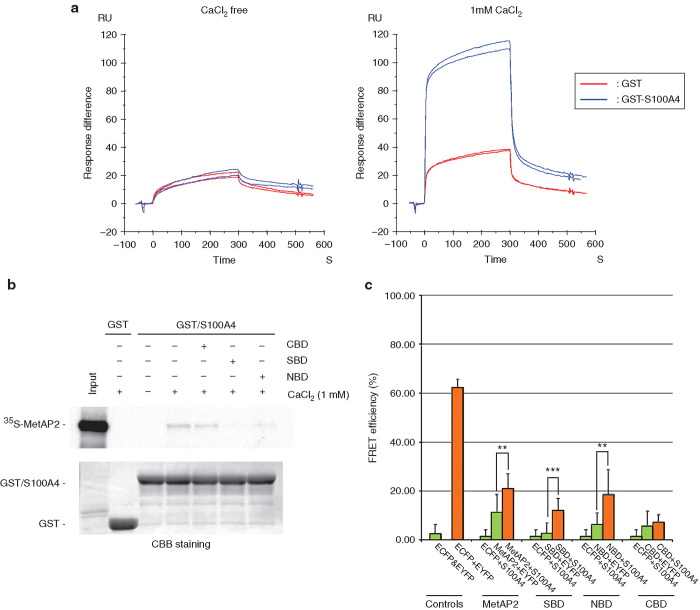
Interaction of S100A4 with the NBD peptide. (**a**) SPR analyses. The SBD peptide was injected over the captured GST-S100A4 and GST in the presence or absence of 1 mmol/l CaCl_2_. The data shown are from a duplicated experiment. (**b**) *In vitro* binding assay. MetAP2 was incubated with either GST or the GST/S100A4 fusion protein in the presence of 1 mmol/l EDTA or 1 mmol/l CaCl_2_. Peptide was added at a 25-fold molar excess of the amount of MetAP2. (**c**) FRET analysis. The graph shows FRET efficiencies (%) calculated from the acceptor-photobreaching analyses in HeLa cells expressing indicated protein pairs. Green bars are negative controls, while orange bars are the positive control (tandem-linked ECFP+EYFP) and experimental groups. *n* = 8–11. **P < 0.01; ***P < 0.001.

To obtain direct evidence that the SBD binds to Ca^2+^-bound S100A4 and hampers the interaction with MetAP2, a peptide, SBD, consisting of 60 amino acid residues spanning from Arg170 to Ala229 of MetAP2 was chemically synthesized and its effect was analyzed by Surface Plasmon Resonance analyses. As shown in Figure 2a, the binding of SBD to S100A4 occurred only in the presence of Ca^2+^. To narrow down the region necessary for blocking S100A4-MetAP2 binding, we synthesized two additional peptides, NBD (N-terminal 39 amino acid sequence of SBD, from Arg170 to Ileu208) and CBD (C-terminal 38 amino acid residues of SBD from Thr192 to Ala229) and tested the effect of these peptides on S100A4-MetAP2 interaction by *in vitro* GST pull-down (binding) assays. Consistent with a previous finding,^24^ S100A4 bound to MetAP2 in a Ca^2+^-dependent manner and the SBD peptide efficiently blocked the interaction between S100A4 and MetAP2 (Figure 2b). The NBD peptide, but not the CBD peptide, inhibited the binding as efficiently as SBD. These results indicate that the NBD peptide harbors an essential binding sequence to Ca^2+^-bound S100A4 and interferes with the interaction of both proteins.

The interaction of S100A4 with MetAP2 with the synthetic peptides in the cell was further examined using Fluorescence Resonance Energy Transfer (FRET) analyses. Figure 2c shows the FRET efficiencies between the pair of proteins indicated. The FRET efficiency (%) calculated in HeLa cells expressing the positive control (tandem-linked EYFP+ECFP protein) was between 50–60%, while that of a negative control (expressing unlinked ECFP and EYFP) was between 3–5%. Those values were similar to the values reported previously,^29^ confirming the reliability of the present FRET measurements. The calculated FRET efficiency (%) between ECFP-MetAP2 and EYFP-S100A4 (orange bar) was significantly higher than that of the negative controls (green bars: ECFP/EYFP-S100A4 and ECFP-MetAP2/EYFP). In addition, the FRET efficiencies for ECFP-SBD/EYFP-S100A4 and ECFP-NBD/EYFP-S100A4, but not for ECFP-CBD/EYFP-S100A4 (orange bars), were significantly higher than those in respective negative controls (green bars). Significantly elevated FRET efficiency could be observed only when two dye molecules, ECFP and EYFP, were positioned closely. Consequently, those data indicate that S100A4 indeed interacts with full-length MetAP2 and the SBD and NBD peptides, but not with the CBD peptide.

To examine if there are any proteins with amino acid sequence similar to NBD, we performed protein-protein BLAST search. We found that the amino acid sequence of NBD did not share a sequence on other proteins, indicating the specificity of the NBD peptide.

### Delivery of the NBD peptide into endothelial cells

**Figure 3:**
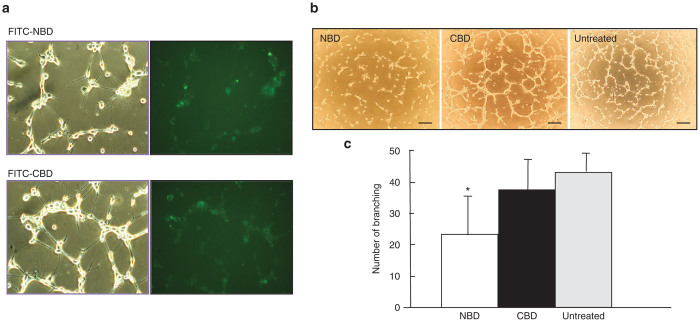
Antiangiogenic activity of the NBD peptide. (**a**) Delivery of the peptides. MSS31 cells were incubated with atelocollagen alone or FITC-labeled NBD or CBD peptide/atelocollagen for 4 hours, cultured for another 6 hours, and then observed under a fluorescent microscope. Left, phase contrast. Right, fluorescence. Scale bars: 50 μm. (**b**) Capillary formation assays. MSS31 cells were incubated with the peptide/atelocollagen complexes and then the cells were inoculated onto Matrigel in the presence of 50 ng/ml HGF. Capillary formation was assessed 16 hours after the Matrigel culture. Scale bars: 50 μm. (**c**) Capillary formation of **b** was measured by counting the number of branching (*n* = 6). *P < 0.01.

The above results indicate that the NBD and the SBD peptide can compete S100A4 effector proteins out from the hydrophobic binding pocket of Ca^2+^-bound S100A4.^28^ Because both the NBD and the SBD peptides contain the amino acid sequence essential for binding with S100A4 but with the former showing higher solubility than the latter, we used the NBD peptide for subsequent studies. To deliver the peptides into MSS31 cells, we selected atelocollagen as a delivery agent.^27,30^ When FITC-labeled NBD or CBD peptide/atelocollagen complexes were added to cultures of MSS31 cells, both peptides complexed with atelocollagen were well incorporated into the cells to the same extent (efficiency of more than 70%) (Figure 3a), while the FITC-labeled peptide alone was not incorporated into the cells (Supplementary Figure S2). Assessment of cell fluorescence revealed the uptake of the peptides by the cells by 10 hours after the start of incubation, showing more than 70% of cells positive for fluorescence. No apparent cytotoxic effect was observed in the presence of atelocollagen. When the peptide-loaded cells were cultured on Matrigel, FITC-positive cells were still observed after 18 hours. These results clearly indicate that the synthetic peptides complexed with atelocollagen were efficiently delivered into the cells. There was no change in the expression level of S100A4 in the peptide-loaded cells (Supplementary Figure S3).

### Antiangiogenesis activity of the NBD peptide *in vitro* and *in vivo*

Next, the effect of the NBD peptide on tube formation was examined. The NBD peptide/atelocollagen complex severely impaired tube formation, while the control CBD peptide showed no inhibitory activity (Figure 3b,c). A kinetic analysis revealed that the inhibition of capillary formation by the NBD peptide was apparent by 6 hours after peptide loading (Supplementary Figure S4). The peptide/atelocollagen complex did not show any cytotoxic effect on MSS31 cells, as assessed by caspase-3 activation and trypan blue dye exclusion test (Supplementary Figure S5)

**Figure 4:**
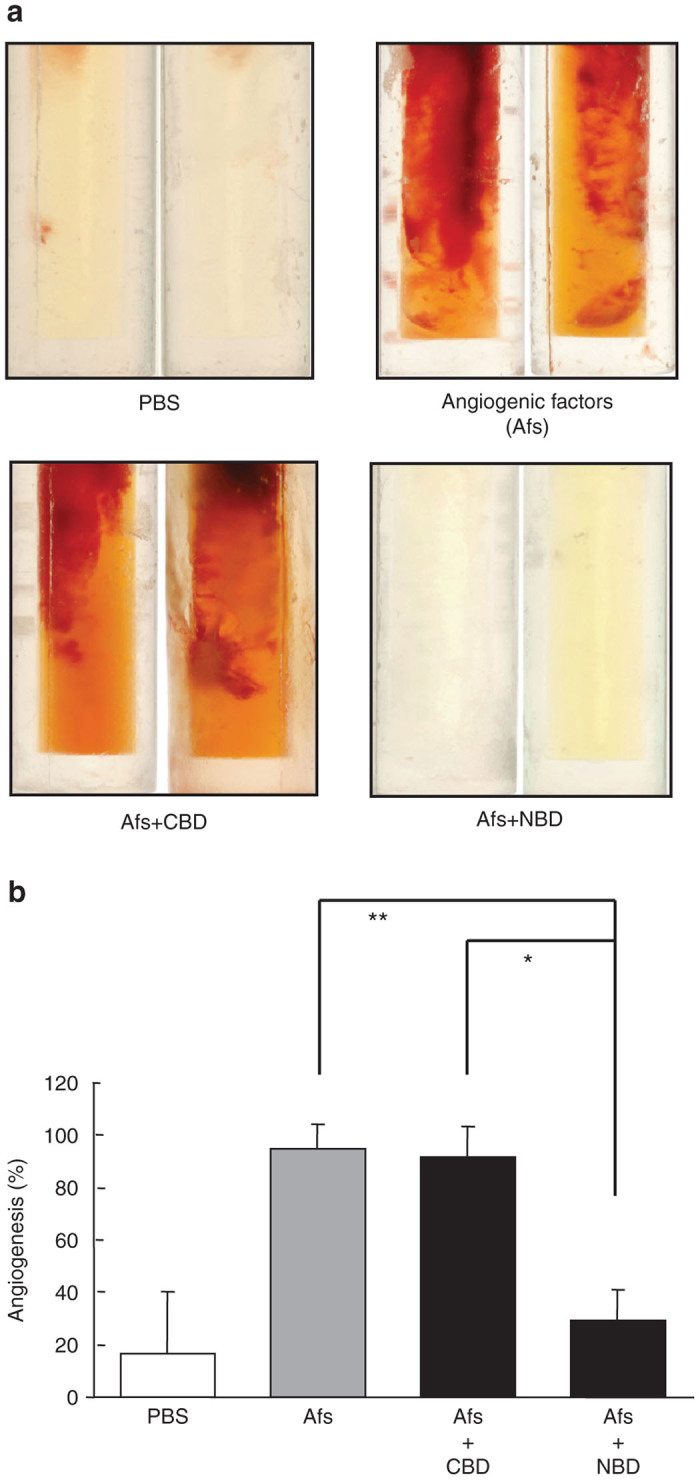
Inhibition of *in vivo* angiogenesis by the NBD peptide. (**a**) DIVAA assay. Angioreactors containing PBS, angiogenic factors (Afs), or Afs plus the CBD or the NBD peptide together with atelocollagen were subcutaneously implanted. (**b**) Angiogenesis was assessed using the index described in the Materials and Methods section. Data represent the mean ± SD (*n* = 3), *P* < 0.01 versus Afs and *P < 0.05 versus Afs and control peptide.

The effect of the NBD peptide was also examined using an *in vivo* angiogenesis assay in nude mice.^31^ Induction of new blood vessel formation was not evident in the angioreactor containing PBS alone. By contrast, angiogenic factors (FGF-2 and VEGF) significantly induced the development of new blood vessels. Inclusion of the NBD peptide potently suppressed the angiogenic factors-induced angiogenesis (*P* < 0.01) while the CBD peptide had no effect (Figure 4a,b). These results clearly indicate that the NBD peptide has an antiangiogenic activity.

### Inhibition of tumor angiogenesis and growth by the NBD peptide

**Figure 5:**
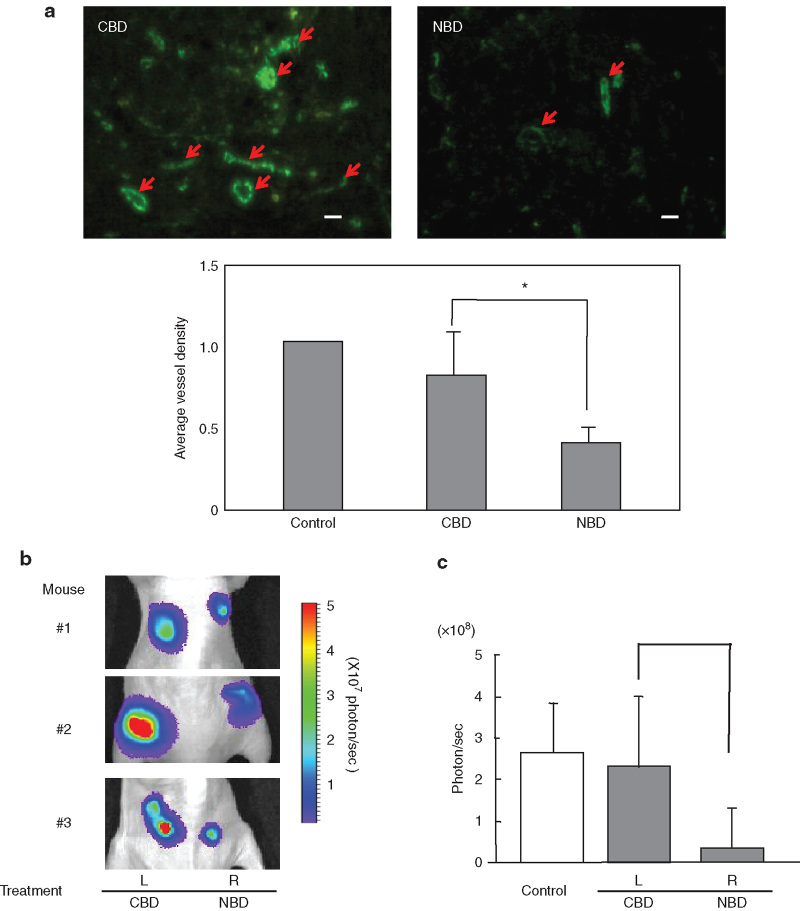
Inhibition of tumor angiogenesis and tumor growth of human prostate cancer cells by the NBD peptide. The peptide/atelocollagen was injected into the subcutaneous tumors on day 3 after inoculation of PC-3M-Luc cells. Atelocollagen alone was injected as controls. (**a**) Microvessel density. Anti-CD31-antibody staining was used to detect angiogenesis. Arrows show microvessels. The images were digitally processed for assessment of angiogenesis. (**b**) Inhibition of tumor growth by peptide delivery *in vivo*. Representative bioluminescent images of nude are shown. L, tumors treated with the CBD peptide; R, tumors treated with the NBD peptide. (**c**) The photon count of the bioluminescence is shown. Data represent the mean ± SD. The total number of animals is 7 in two independent experiments.

To assess the antiangiogenic and tumor growth-inhibitory abilities of the NBD peptide in an animal model, PC-3M-Luc cells were inoculated into athymic nude mice. When tumors reached 5 mm in diameter, the NBD or the CBD peptide/atelocollagen complex was intratumorally administered once. Angiogenesis in the tumors treated with the NBD peptide was severely impaired, while the control CBD peptide showed no inhibitory effect (Figure 5a, arrow). Monitoring of tumor growth by *in vivo* bioluminescence imaging on day 14 after the administration of the peptide/atelocollagen complex (Figure 5b,c) showed a significant inhibition of the growth of tumors treated with the NBD peptide compared to those treated with atelocollagen alone (control) and the CBD peptide (number of animals in each group = 7, *P* < 0.05). Treatment of PC-3M-Luc cells with the NBD peptide *in vitro* resulted in a comparable growth rate to that of untreated cells (Supplementary Figure S6), indicating that the NBD peptide itself does not affect the growth of the tumor cells. Thus, the atelocollagen-mediated delivery of the NBD peptide potently inhibited tumor angiogenesis and thereby resulted in tumor growth inhibition.

### Effect of the NBD peptide on NMIIA phosphorylation and filament assembly

**Figure 6:**
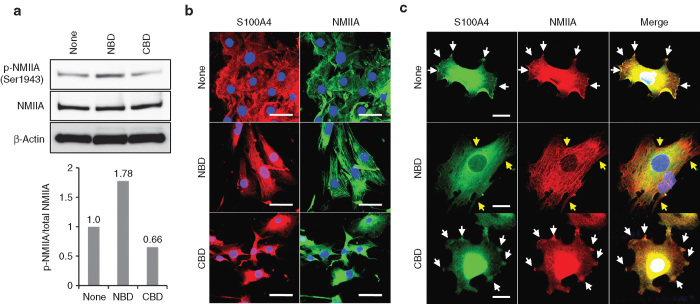
Stimulation of NMIIA phosphorylation and filament assembly by the NBD peptide. MSS31 cells incubated with atelocollagen alone or the peptide/atelocollagen complexes were subjected to western blot analysis and immunofluorescent staining. (**a**) Western blot analysis of NMIIA phosphorylation. Total cellular proteins were subjected to immunoblot analysis. The band intensity of p-NMIIA relative to that of total NMIIA was measured using ImageJ software and normalized to control cells. (**b**) NMIIA assembly. MSS31 cells were simultaneously incubated with rabbit polyclonal anti-S100A4 antibody and mouse monoclonal anti-NMIIA antibody followed by Alexa Fluor 488-conjugated goat antirabbit IgG and Alexa Fluor 594-conjugated goat antimouse IgG. Nuclei were stained with DAPI. Scale bars: 50 μm. (**c**) Blockade of S100A4-NMIIA interaction by the NBD peptide. MSS31 cells were immunostained as in **a**. White arrows and yellow arrows indicate colocalized areas and independently localized areas of S100A4 and NMIIA, respectively. Bars: 20 μm.

To gain mechanistic insight into the action of the NBD peptide, we examined its effect on NMIIA phosphorylation and filament assembly, since CK2-mediated phosphorylation and self-assembly of NMIIA have been shown to be inhibited by S100A4 binding.^32,33^ As expected, the peptide enhanced Ser1943 phosphorylation of NMIIA (Figure 6a) and stimulated filament assembly in MSS31 cells (Figure 6b). NMIIA filaments were evenly distributed throughout the cells and showed no polarization. These results suggest that the NBD peptide preferentially blocks S100A4 binding to NMIIA filaments, resulting in stimulation of the NMIIA phosphorylation and, as a result, stabilization of the filaments. Supporting this, a close look at the colocalization of S100A4 and NMIIA revealed that they were colocalized in the lamellipodia and the leading edges of the control and the CBD-treated cells whereas the majority of S100A4 was not colocalized with NMIIA filaments in the NBD-treated cells (Figure 6c).

### Effect of the NBD peptide on the formation of focal adhesions and FAK phosphorylation

**Figure 7:**
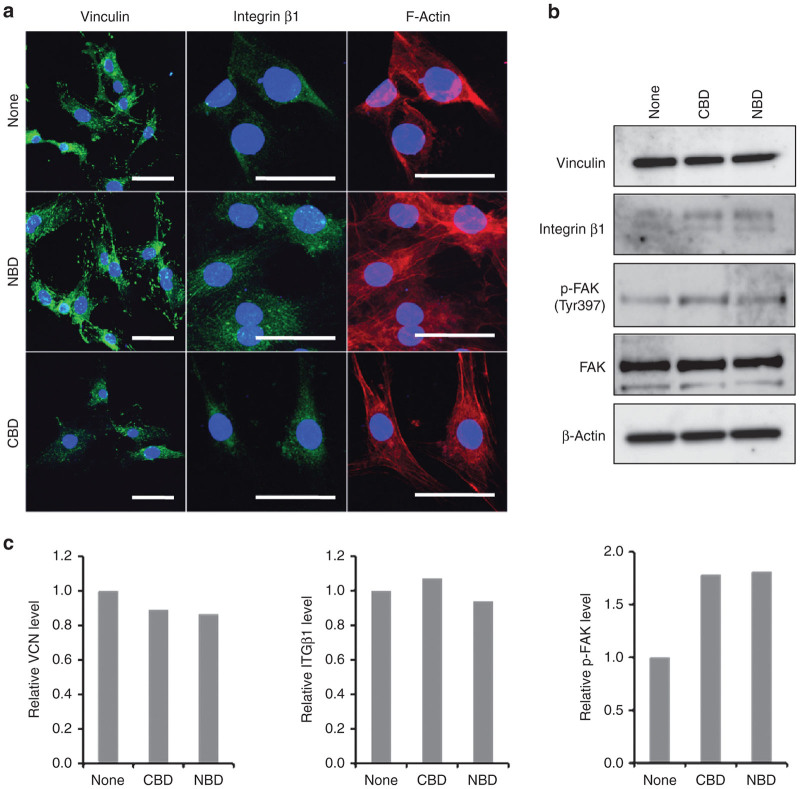
Stimulation of the formation of focal contacts by the NBD peptide. MSS31 cells incubated with atelocollagen alone or the peptide/atelocollagen complexes were subjected to immunofluorescent staining and western blot analysis. (**a**) Formation of focal adhesions. The cells were immunostained with antivinculin or anti-integrin β1 antibody followed by Alexa Fluor 488-conjugated secondary antibody. F-actin was visualized with Atto 647N-Phalloidin. Nuclei were stained with DAPI. Scale bars: 50 μm. (**b**) Western blot analysis of focal adhesion proteins. Total cellular proteins were subjected to immunoblot analysis. (**c**) Relative expression level of vinculin (VCN), integrin β1 (ITGβ1) and p-FAK. The band intensity of VCN and ITGβ1 and p-FAK relative to that of β-actin and total FAK, respectively, was measured using ImageJ software and normalized to control cells.

Immunofluorescent staining for vinculin and integrin β1 and phalloidin staining for F-actin were also carried out to examine the effect of the NBD peptide on the formation of focal adhesions in MSS31 cells. The cells showed a greater number of vinculin- and integrin β1-containing focal adhesions per cell than the untreated and the CBD peptide-treated cells (Figure 7a). Formation of actin filaments was also prominent in the cells. However, we found no significant increase in the phosphorylation of Tyr397 of FAK (focal adhesion kinase) in the NBD-treated cells compared to the control and the CBD peptide-treated cells (Figure 7b). It should be noted that focal adhesions were distributed uniformly in the NBD-treated cells. Immunoblot analyses using total cell lysates showed no significant difference in the expressions of vinculin, integrin β1 and FAK between control cells and the NBD peptide-treated cells (Figure 7b,c), indicating that a stimulated focal adhesion complex assembly does not require *de novo* synthesis of these proteins.

### Effect of the NBD peptide on cell cycle progression

**Figure 8:**
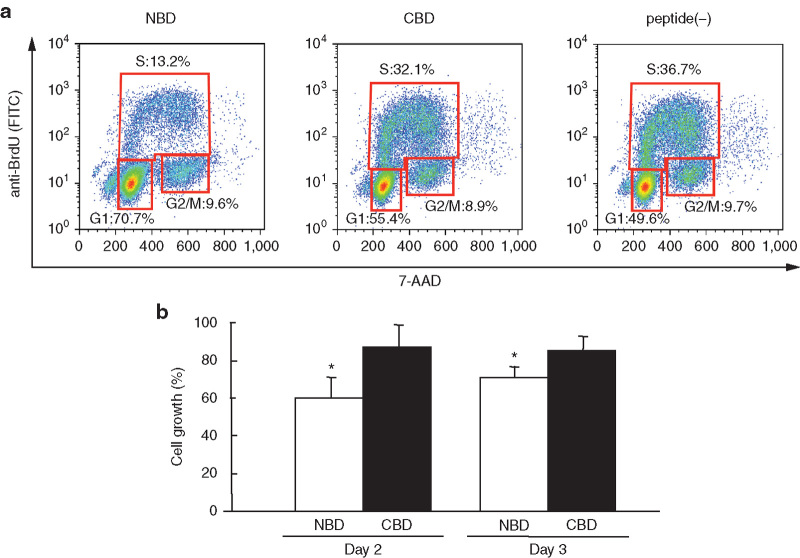
Inhibition of cell cycle progression and cell growth by the NBD peptide. MSS31 cells were treated with atelocollagen alone or the NBD or the CBD peptide/atelocollagen complex *in vitro*. (**a**) Cell cycle progression. The cells were labeled with BrdU and then subjected to flow cytometry. (**b**) Cell growth. The values represent cell growth rate relative to that of control cells that is set to 100%. The NBD peptide inhibited the growth of MSS31 cells compared to the control cells (atelocollagen alone). Data represent the mean ± SD (*n* = 4), *P* < 0.01.

Since the overexpression of the SBD in MSS31 cells caused DNA synthesis inhibition, we examined the effect of the NBD peptide on cell cycle progression by flow cytometry after treating the cells with various concentrations of the synthetic peptides (0–1.0 μg/well) complexed with atelocollagen for 9 hours. As shown in a representative study (Figure 8a), the percentage of cells in G_1_ phase increased from 49.6% in the absence of the peptide to 70.7% in the presence of the NBD peptide. Simultaneously, a decrease in the percentage of cells in S phase from 36.7% in the absence of the peptide to 13.2% in the presence of the NBD peptide was observed. The G_2_/M phase was not significantly affected by the NBD peptide. In contrast, in the case of the CBD peptide, only a marginal effect on cell cycle progression could be detected. These results indicate that the NBD peptide, but not the CBD peptide, leads to cell cycle arrest in the G_1_/S phase in MSS31 cells. This was further corroborated by the fact that the NBD peptide significantly inhibited the growth of MSS31 cells (Figure 8b).

### Effect of the NBD peptide on the expression of angiogenesis-related genes

We have recently demonstrated that knockdown of S100A4 in MSS31 cells resulted in inhibition of the expression of proangiogenic genes such as *retn*, *fgf18*, *aqp1*, *map3k5*, *thy1*, *foxo6*, and *hs6st1* and stimulation of the expression of antiangiogenic genes including *cdkn1a*, *thbs1*, and *spry4*.^34^ Therefore, an alternative action mechanism of the NBD peptide would be the modulation of the expression of these angiogenesis-related genes. However, we could not detect any alteration of the expression of these genes when assessed by qRT-PCR (Supplementary Figure S7).

## Discussion

In this study, we demonstrated that the NBD peptide complexed with atelocollagen, which is a highly purified type I collagen,^34,35^ potently inhibits *in vitro* tube formation of MSS31 cells and angiogenesis in a directed *in vivo* angiogenesis assay. Importantly, the peptide/atelocollagen complex caused a significant reduction in tumor angiogenesis and tumor size after a single intratumor administration, indicating a long-lasting effect of the complex. It is probable that the NBD/atelocollagen complex is persistently incorporated into cells *in vivo* over a long period.

The NBD peptide was designed to inhibit S100A4-MetAP2 interaction, which was confirmed by Surface Plasmon Resonance analysis, in an *in vitro* pull-down assay and FRET analysis. Since the Ca^2+^-bound form of S100A4 opens up a hydrophobic target-binding pocket to which many other binding partners also bind,^28^ the peptide may hinder the binding of these effector proteins except those with higher affinity to S100A4 than the peptide, resulting in appearance of a variety of phenotypes. We then sought mechanisms underlying inhibition of angiogenesis by the NBD peptide. First, we found increased NMIIA filament assembly and Ser1943 phosphorylation of NMIIA in the NBD peptide-loaded cells. S100A4 has been shown to interact with the C-terminal end coiled-coil of the NMIIA and directly inhibit the formation of NMIIA filaments, resulting in increases of cell migration.^32,33^ Phosphorylation of Ser1943 of NMIIA protects against S100A4-induced filament disassembly.^7,8,32^ Thus, it is likely that the NBD peptide stabilizes myosin filaments by directly inhibiting the ability of S100A4 to disassemble the filaments through increasing Ser1943 phosphorylation. Second, we noticed the formation of a larger number of focal adhesion sites per cell in the NBD peptide-loaded cells than in the control cells. Since activation of FAK by autophosphorylation of Tyr397 in response to integrin clustering enhances turnover of focal adhesions and cell migration,^36,37^ we also examined FAK phosphorylation. However, the NBD peptide did not affect Tyr397 phosphorylation. It should be noted that both myosin filaments and focal adhesions were distributed uniformly in the NBD-treated cells, unlike motile cells in which focal adhesions are localized at the leading edge and myosin filaments are distributed peripherally.^36–38^ It is plausible, therefore, that the NBD peptide causes unpolarized NMIIA filament assembly and formation of focal adhesions. Third, we found that the NBD peptide led to cell cycle arrest in MSS31 cells. This observation is consistent with the results of overexpression of the SBD in the cells. Although we cannot presently explain the mechanism of the cell cycle arrest, we presume that it is attributed to the blockade of S100A4-MetAP2 interaction, because knockdown of S100A4 by siRNA suppresses endothelial cell growth^27^ and MetAP2 is a target of antiproliferative, antiangiogenic agents.^20–22^ Alternatively, it might also be due to the stabilization of NMIIA, which has recently been reported to be a tumor suppressor,^39^ since CK-2 mediated phosphorylation of NMIIA retained in a suppressed state in MSS31 cells, when treated with the NBD peptide, is converted to an activated state and results in G1-arrest of the cell cycle. Collectively, it is probable that formation of inflexible NMIIA filaments caused by Ser1943 phosphorylation, along with enhanced focal adhesion anchoring forces, causes suppression of cell migration and cell cycle arrest, consequently leading to inhibition of angiogenesis. Supporting this notion, knockdown of S100A4 in MSS31 cells by siRNA enhanced cell adhesion and reduced cell migration.^27^ Furthermore, a recent report demonstrated that S100P, an S100 family member, dissociates NMIIA filaments, and knockdown of NMIIA dramatically decreased the number of focal adhesions and eventually reduced adhesion and increased cell motility in HeLa cells.^40^ Collectively, these results suggest that the antiangiogenic effect of the NBD peptide is attributed to the inhibition of cell growth and cell motility of MSS31 cells.

We demonstrated that the expressions of several proangiogenic genes are suppressed while those of some antiangiogenic genes are stimulated in S100A4-depleted MSS31 cells.^27^ However, we could not detect any alteration of the expression of these genes in the NBD peptide-treated cells. Since no apparent change in the expression level of S100A4 could be observed in the cells with the NBD peptides transduced, it may be true that the NBD peptide is not directly involved in controlling angiogenic-related genes. From these results, we presently presume that S100A4 is involved in two pathways controlling angiogenesis: one affects the expression of angiogenesis-related genes and the other does not, by using different effector proteins. It is possible that the former pathway contains S100A4 localized in the nucleus,^41,42^ to which the incorporated NBD peptide cannot reach, and that the latter comprises cytoplasmic S100A4. Depletion of S100A4 by siRNA may make both pathways inactive, and the NBD peptide may suppress only the latter pathway but that the suppression is sufficient to inhibit tube formation. Alternatively, although more likely, the inability of the NBD peptide to alter the angiogenesis-related gene expression can simply be attributed to the multifunctionality of S100A4. The NBD peptide may not be able to prevent the interaction between S100A4 and the effector protein(s) that leads to the alteration of gene expression. To finely resolve the action mechanism of the NBD peptide, further studies are required.

Curiously, the NBD peptide did not influence the proliferation of PC-3 cells, irrespective of the identical amino acid sequence to the corresponding region of human MetAP2. Although we have no direct evidence, this may attribute to the status of the tumor suppressor p53 protein; MSS31 cells have wild-type p53 whereas PC-3 cells are p53-null.^43^ It has been shown that S100A4 interacts with p53 and this interaction modulates p53 transactivation.^10^ The consequence of this modulation is complicated by the fact that several of the p53-responsive genes are prosurvival whereas others are proapoptotic.^10^ It seems likely that the balance of the expression of the prosurvival genes and the proapoptotic genes seems to depend on cell types and culture conditions.^17^ Therefore, it is possible that S100A4 functions as a prosurvival, growth-promoting mediator in MSS31 cells, but it hardly affects the expression of the p53-related genes in p53-null PC-3 cells. This may be the reason why the NBD peptide can inhibit the growth of MSS31 cells but not of PC-3 cells. Further studies are required to address this issue.

In this study, we focused on S100A4-MetAP2 interaction in MSS31 cells. However, the cells also express S100A6 and perhaps other S100 family proteins except S100A1.^24^ Although there is no data about the interaction between S100 family proteins and MetAP2, we cannot exclude the possibility that the effect of NBD might be combination of inhibition of multiple S100 proteins.

In general, the major disadvantages of the peptide drugs are poor stability, poor membrane permeability, and rapid clearance in tissues.^44^ The NBD peptide also has these disadvantages. In addition, as stated above, S100A4 interacts with several binding partners, and blocking one of the interactions by the peptide would leave unaffected the other interaction, which is of importance from a mechanistic and therapeutic viewpoint. In that sense, the NBD peptide drug strategy may be less efficient than the S100A4 siRNA strategy *in vivo*.^27^ However, as demonstrated in this study, the usage of atelocollagen can improve stability, membrane permeability, and clearance of the peptide. Furthermore, a single injection of the NBD peptide/atelocollagen resulted in the inhibition of tumor angiogenesis and tumor growth. This simplicity of use may be an advantage of the NBD peptide/atelocollagen complex when compared with the S100A4 siRNA strategy.^27^

In conclusion, the present results show that the NBD peptide has a potent inhibitory effect on tumor angiogenesis. At least in the mouse model, the peptide-based drugs against S100A4 have a potential as a novel antiangiogenic therapeutic agent, which is involved in starvation tactics-type anticancer drugs pioneered by Folkman,^45^ for both S100A4-positive cancers and -negative cancers. Further efforts to develop S100A4 inhibitors in the form of peptide drugs or small-molecule drugs may be promising for the development of effective anticancer agents.

## Materials and Methods

### Cell culture

Mouse endothelial MSS31 cells,^27^ Phoenix cells, PtG-S2 cells (originated from HT1080), HeLa cells, and human prostate carcinoma cells expressing a firefly luciferase gene named PC-3M-Luc (Caliper Life Sciences, Hopkinton, MA)^27^ were cultured in DMEM supplemented with 10% FBS.

### Construction and expression of recombinant retrovirus vectors

cDNA fragments encoding amino acids 1–169 [MetAP2(1–169)] or 1–229 [MetAP2(1–229)] of MetAP2 were PCR amplified and inserted into the multicloning site (SalI/NotI site) of the pRX-puro vector (Supplementary Figure S1). High titer recombinant VSV-G-pseudotyped retroviruses carrying MetAP2(1–169) or MetAP2(1–229) were produced by the standard packaging method^46^ and used to infect MSS31 cells. Immunoblot analysis was carried out to confirm the expression of both recombinant proteins using a rabbit polyclonal anti-MetAP2 antibody (a gift of Dr T Kanno, National Institute of Infectious Diseases, Tokyo) and alkaline phosphatase-conjugated secondary antibody.

### DNA synthesis

DNA synthesis was quantified by measuring bromodeoxyuridine (BrdU) incorporation using the Cell Proliferation ELISA BrdU kit (Roche Applied Science, Penzberg, Upper Bavaria, Germany). MSS31 cells cultured for 24 hours in a medium containing 0.2% serum were stimulated with a medium containing 10% FBS and 10 μmol/l BrdU for 6 hours. The cells were harvested and BrdU uptake was measured according to the manufacturer’s protocol. Briefly, the cells were fixed, denatured, and incubated with the monoclonal anti-BrdU-peroxidase antibody. The immune complexes were then detected by the subsequent reaction with tetramethyl-benzidine. The reaction product was quantified by measuring the absorbance (OD450) using a scanning multiwell spectrophotometer.

### Peptide synthesis

Peptides were synthesized by the solid phase method with a Pioneer peptide synthesis system (Applied Biosystems, Foster City, CA) and purified by reverse-phase high performance liquid chromatography on a C18 column. The synthesized peptides were (i) SBD, corresponding to MetAP2 (170–229), (ii) NBD, corresponding to MetAP2 (170–208), and (iii) CBD, corresponding to MetAP2 (192–229). FITC-labeled-NBD and -CBD peptides were synthesized at Bio-Synthesis (Lewisville, TX) and purified by preparative reverse-phase HPLC (>95% pure).

### Surface plasmon resonance analysis

The binding of the SBD peptide to S100A4 was examined by Surface plasmon resonance (SPR) with a Biacore 3000 biosensor and GST Capture kit (GE Healthcare, Little Chalfont, UK) according to the manufacturer’s instructions. Goat anti-GST antibody was immobilized on two flow cells of the sensor chip (CM-5) using a standard amine coupling procedure, and then recombinant GST-S100A4 or GST as a negative control was captured. The SBD peptide (1 mmol/l) in HBS-P running buffer (10 mmol/l Hepes, pH 7.4, 150 mmol/l NaCl, 0.005% P20) in the presence or absence of 1 mmol/l CaCl_2_ was injected over two flow cells of the chip at a flow rate of 10 ml/minute at 25 °C for 5 minutes. The response from the control surface was subtracted, and the data plotted as Resonance Units (RU).

### *In vitro* binding assay

*In vitro* transcription and translation of MetAP2 cDNA subcloned into pBluescript (SK-) were carried out with the TNT quick coupled transcription/translation system (Promega, Fitchburg, WI) using [^35^S]methionine (>1,000 Ci (37 TBq)/mmol) as a tracer amino acid. The yield of MetAP2 protein was determined as described in the TNT protocol with minor modifications. The binding assays for [^35^S]MetAP2 and GST or GST/S100A4 fusion proteins were carried out in the presence of either 1 mmol/l CaCl_2_ or 1 mmol/l EDTA as described previously.^24^

### Fluorescence resonance energy transfer analyses

Fluorescence resonance energy transfer (FRET) analysis is a method to evaluate the distance between two donor- and acceptor-dye-molecules. FRET is a transfer of excitation energy from donor to acceptor molecule (calculated as FRET efficiency (%)), which occurs effectively only when the distance between the two dye-molecules is less than 10 nanometers, *i.e.*, they are most likely physically interacting and in a single complex. We employed FRET analyses in order to observe the interaction between S100A4 and MetAP2 in live cells. As donor and acceptor molecules, we selected the most commonly used pair, ECFP and EYFP, respectively. For expression of EYFP-tagged S100A4 and ECFP-tagged MetAP2, the coding sequences of S100A4 and full-length MetAP2 lacking the initiation methionine were subcloned into pEYFP-C1 and pECFP-C1 (Clontech Laboratories/Takara Bio, Shiga, Japan), respectively. The sequences corresponding to the peptides SBD, NBD and CBD were also subcloned into the pECFP-C1 vector. The FRET pairs of EYFP-S100A4 and ECFP-MetAP2, and those of EYFP-S100A4 and each ECFP-MetAP2 peptide (SBD, NBD, or CBD) were cotransfected into HeLa cells by Lipofectamine2000 (Invitrogen, Life Technologies). In order to eliminate nonspecific interaction between S100A4 and ECFP; MetAP2 and EYFP; EYFP and ECFP; we prepared series of HeLa cells expressing following combination of proteins as negative controls: ECFP/EYFP, ECFP/EYFP-S100A4, ECFP-MetAP2/EYFP, ECFP-SBD/EYFP, ECFP-NBD/EYFP, and ECFP-NBD/EYFP. As a positive control, the tandem-linked ECFP+EYFP protein was expressed by transfecting ECFP+EYFP-pCDNA6myc-His B to HeLa cells. After transfection, HeLa cells were further incubated for 24 hours prior to FRET analyses. In order to calculate the FRET efficiency (%), the acceptor-photobleaching method was employed following the protocol described by Liu *et al*.^29^ with minor modifications. For imaging of ECFP and EYFP and photo-breaching in live HeLa cells, LSM710 confocal microscope (Carl Zeiss AG, Jena, Germany) was used. Expression of the respective plasmids was confirmed by western blotting.

### Peptide loading and capillary morphogenesis

A mixture of atelocollagen (80 μg/ml, AteloGene, Koken, Tokyo, Japan) and FITC-labeled or unlabeled synthetic peptides (NBD, CBD) (1 mg/ml) (the size of atelocollagen/peptide complex was around 175–200 nm in diameter as measured by nanoparticle analyzer) was added into a culture of MSS31 cells at 8 × 10^4^ cells/2 ml 10% FBS-DMEM/well in six-well plates. The final peptide concentration was 5 μg/well/8 × 10^4^ cells. Atelocollagen alone was used as a control. The cells were cultured in serum-free DMEM for 4 hours followed by a change into 3% FBS-containing DMEM. For analysis of peptide uptake, cells were cultured for another 6 hours and then analyzed under a fluorescent microscope. For capillary morphogenesis analysis, the transfected cells were inoculated onto 24-well Matrigel plates (Geltrex: Reduced Growth Factor Basement Membrane Matrix, 63 μl/cm^2^, Life Technologies, Carlsbad, CA) at a cell density of 4 × 10^4^ cells/cm^2^ and cultured in the presence of 50 ng/ml HGF (PeproTech, Rocky Hill, NJ). Capillary formation was assessed 16 hours after the Matrigel culture.

### Flow cytometric analysis

MSS31 cells treated with 1.0 μg/ml of the NBD peptide or the CBD peptide/atelocollagen were labeled with 10 μmol/l BrdU for 6 hours. Atelocollagen alone was used as a control. Flow cytometric analysis was carried out using the FITC BrdU Flow Kit (BD Biosciences Pharmigen, San Diego, CA) following the manufacturer’s instructions.

### *In vivo* angiogenesis assay

A mixture of atelocollagen (80 μg/ml in PBS(-)) and the synthetic peptides NBD and CBD (1 mg/ml) was prepared by incubation under rotation in 20-mm sterile surgical silicone tubes (angioreactor) of a DIVAA kit (Trevigen, Gaithersburg, MD) which were filled with Matrigel with or without angiogenic factors (bFGF, VEGF and heparin). These were incubated at 37 °C for 1 hour for gel formation, and implanted subcutaneously into the dorsal flank of BALB/c nude mice (BALB/cAJcl-*nu*/*nu,* females, 7-weeks-old; CLEA Japan, Tokyo, Japan). After 15 days, angioreactors were collected, and the level of angiogenesis was determined using four angiogenesis indices of 0, 1, 2, and 3, according to a modification of the method previously described.^47^ Grade 0 indicated no angiogenesis, while grade 3 indicated the highest degree of angiogenesis. Angiogenesis was indicated by the presence of grades 2 and 3. Angiogenesis (%) was calculated by the following equation: % = (number of angioreactors showing angiogenesis)/(total number of angioreactors) × 100. Data were expressed as the mean ± SD. Statistical analyses were performed using the Student’s *t*-test.

### *In vivo* bioluminescent imaging of xenografted tumors

Animal experiments were performed in compliance with the guidelines of the Institute for Laboratory Animal Research at the National Cancer Center Research Institute. Eight- to 9-week-old male athymic nude mice (CLEA Japan, Osaka) were used for all the experiments. Anesthetized animals were subcutaneously injected with 3 × 10^6^ human prostate cancer cells expressing firefly luciferase (PC-3M-Luc cells, Xenogen) in 100 μl sterile Dulbecco’s PBS.^33^ For *in vivo* imaging, mice were administered d-luciferin (150 mg/kg, Promega) by intraperitoneal injection. Ten minutes later, photons from animal whole bodies were counted using the IVIS imaging system (Caliper Life Sciences) according to the manufacturer’s instructions. Data were analyzed by living image 2.50 software (Caliper Life Sciences).

### *In vivo* peptide delivery

The peptide/atelocollagen complex was prepared by mixing equal volumes of atelocollagen (0.1% in PBS at pH 7.4) and peptide solution (1 mg/ml) with rotation for 30 minutes at 4 °C. The final concentration of atelocollagen was 0.5%. When a tumor reached 5 mm in diameter, individual mice were injected with 100 μl of atelocollagen containing 100 μg of the synthetic peptide NBD or CBD by intratumoral injection. Atelocollagen alone was injected as a control. Three days after peptide injection, tumors were dissected and subjected to angiogenesis analysis. Tumor growth was assessed through peptide/atelocollagen complex administration to mice 3 days after the subcutaneous inoculation of PC-3M-Luc cells. Mice were monitored by bioluminescent imaging 14 days after the injection of the peptide/atelocollagen complex. Tumors were subjected to preparing specimens of fresh frozen tissue for studying angiogenesis.

### Angiogenesis analysis in tumors

Frozen tumor tissues embedded in a tissue-freezing medium were cut into 5–8 µm-thick sections. For immunohistochemical analysis, tissue sections were fixed in acetone for 10 minutes. The samples were blocked with PBS containing 0.1% bovine serum albumin and 2% human serum and then incubated with a phycoerythrin-conjugated anti-CD31-antibody (BD Pharmingen). The sections were washed three times with PBS and imaged under a fluorescent microscope (ECLIPS1000, Nikon, Tokyo, Japan). Ten random sections were digitally photographed and image-processed. The average pixel density of each peptide-treated tumor section was normalized to the average pixel density of the untreated controls.

### Western blot analysis

MSS31 cells cultured in six-well plates were incubated with 5 μg/well of the NBD or the CBD peptide/atelocollagen complex or atelocollagen alone for 18 hours and were lysed in M-PER Mammarian Protein Extraction Reagent (Thermo Fisher Scientific, Waltham, MA) containing complete, EDTA-free, EASYpack (Roche Applied Science, Penzberg, Upper Bavaria, Germany) and Halt Phosphatase Inhibitor Cocktail (Thermo Fisher Scientific). The lysates was used for immunoblot analysis. Proteins were separated by SDS–PAGE (5–20% gradient gel or 10% gel) under reducing conditions and transferred to an Immobilon-P Transfer Membrane (Merck Millipore, Billerica, MA). The membrane was incubated with first antibodies, washed extensively with TBS-T, and then incubated with species-appropriate HRP-conjugated secondary antibodies. The first antibodies used were rabbit polyclonal anti-S100A4 (Merck Millipore), mouse monoclonal anti-NMIIA antibody (Abcam, Cambridge, MA), rabbit polyclonal antiphospho-NMIIA (Ser1943) antibody (Cell signaling Technology, Danvers, MA), mouse monoclonal antivinculin antibody (Sigma-Aldrich, St Louis, MO), rabbit polyclonal anti-integrin β1 antibody (Merck Millipore), rabbit polyclonal antiphospho-FAK (Tyr397) antibody (Cell Signaling Technology), rabbit polyclonal anti-FAK antibody (Cell Signaling Technology) and mouse monoclonal anti-β-actin antibody (Sigma-Aldrich). Immunodetection was carried out using ECL Plus western blotting Detection Reagent (Amersham Biosciences, Piscataway, NJ).

### Immunofluorescence study

MSS31 cells cultured in six-well plates were incubated with 5 μg/well of the NBD or the CBD peptide/atelocollagen complex or atelocollagen alone for 18 hours in Culture Slides (4 wells, BD Falcon) and were washed three times with DPBS and fixed for 10 minutes at 4 °C with 4% formaldehyde in DPBS. After permeabilizing with 0.5% Triton X-100 in DPBS for 4 minutes, the cells were treated with 3% bovine serum albumin in DPBS containing 0.1% glycine for 1 hour to block nonspecific binding sites. After washing with DPBS, the cells were incubated for 1 hour with primary antibodies, rinsed, and then stained with secondary antibodies. The primary antibodies used were polyclonal anti-S100A4, monoclonal anti-NMIIA antibody, monoclonal antivinculin antibody and polyclonal anti-integrin β1 antibody. The secondary antibody was Alexa Fluor 488-conjugated goat antirabbit IgG (Invitrogen) or Alexa Fluor 594-conjugated goat antimouse IgG (Invitrogen). F-Actin was visualized with Atto 647N-Phalloidin (Sigma-Aldrich). Nuclei were stained with 1 μg/ml DAPI. The slides were mounted with VECTASHIELD Mounting Medium (Vector Laboratories). The cells were observed under a confocal laser scanning microscope (Fluoview FV1000, Olympus, Japan).
