# Supplementary material for: Efficient inhibition of tumor angiogenesis and growth by a synthetic peptide blocking S100A4-methionine aminopeptidase 2 interaction
Source: Mol Ther Methods Clin Dev. 2015 Apr 1;2:15008–. doi: 10.1038/mtm.2015.8 (PMC4445002; doi:10.1038/mtm.2015.8)

Supplementary Information

Fig. S1 Construction and expression of recombinant retrovirus vectors.

(a) pRx-puro vector was used for the backbone vector. (b) cDNA fragments encoding amino acids 1-169 [MetAP2(1-169)] or 1-229 [MetAP2(1-229)] of MetAP2 were PCR amplified and inserted into the multi-cloning site (SalI/NotI site) of the backbone vector.

Fig. S2 Incorporation of the peptides into MSS31 cells.

MSS31 cells were loaded with FITC-labeled synthetic peptides complexed with atelocollagen reagent（AteloGene, KOKEN）. In brief, a mixture of atelocollagen (80 ug/ml) and FITC-labeled synthetic peptides (NBD) (1 mg/ml) was added into culture medium of MSS31 cells at １x10^4 cells/１ ml 10% FBS-DMEM/well in 20-well-plate (a, b). As a control, FITC-labeled NBD alone was used (c, d). For analysis of peptide uptake, the cells were cultured for another 20 h and then assessed for the presence of FITC-labeled cells under a fluorescent microscope (a, c). Approximately, 70% of cells were positive for the uptake of FITC-labeled peptides complexed with atelocollagen. Bar indicates 50 μm.

Fig. S3 S100A4 expression in the peptide-loaded MSS31 cells.

MSS31 cells were incubated with atelocollagen alone or the peptide/atelocollagen complexes. Total cell lysates were prepared and subjected to Western blot analysis with anti-S100A4 antibody. β-Actin was used as a loading control.

Fig. S4 A kinetic analysis of the inhibition of capillary formation by the NBD peptide. MSS31 cells incubated with atelocollagen alone or the NBD peptide/atelocollagen complexes *in vitro* were cultured on Matrigel, and capillary formation was monitored thereafter.

Fig. S5 Effect of the NBD peptide on the death of MSS31 cells.

MSS31 cells were treated with atelocollagen alone (control) or the NBD or the CBD peptide/atelocollagen complex *in vitro*. Cell death was monitored by caspase-3 activation and trypan blue dye exclusion test (Lifetechnologies).

Caspase3/7 assays was performed with Apo-ONE Caspase-3/7 Assay Reagent (Promega). No significant changes are observed.

Fig. S6 Cell growth of PC-3M cells loaded with the NBD peptide. PC-3M-Luc cells were treated with atelocollagen alone or the NBD peptide/atelocollagen complex *in vitro*, and their growth rate was monitored. The values represent cell growth rate relative to that of control cells that is set to 100%. PC-3M-Luc cells loaded with the NBD peptide showed a comparable growth rate to that of the untreated cells.

Fig. S7 qRT-PCR analysis of the angiogenesis-related genes. MSS31 cells were incubated with the peptide/atelocollagen complexes. Isolation of total RNA and subsequent qRT-PCR analysis were carried out as described previously (30). The GAPDH gene was used as an internal control. (a) Proangiogenic genes in NBD and CBD peptides-treated cells. (b) Antiangiogenic genes in NBD and CBD peptides-treated cells.

Supplementary Fig. S1

a

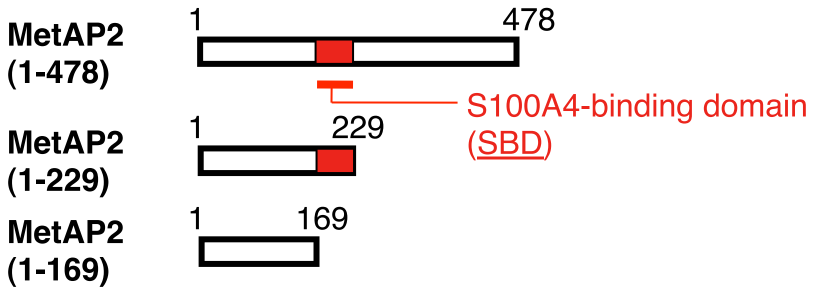

b

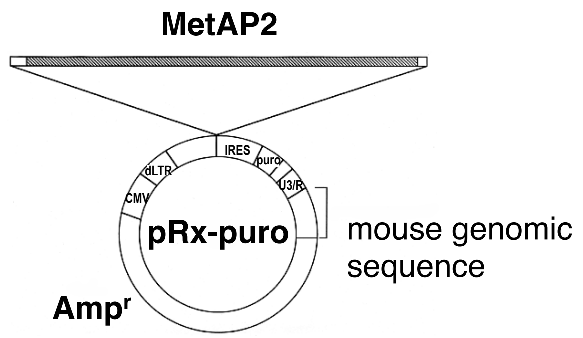

Supplementary Fig. S2

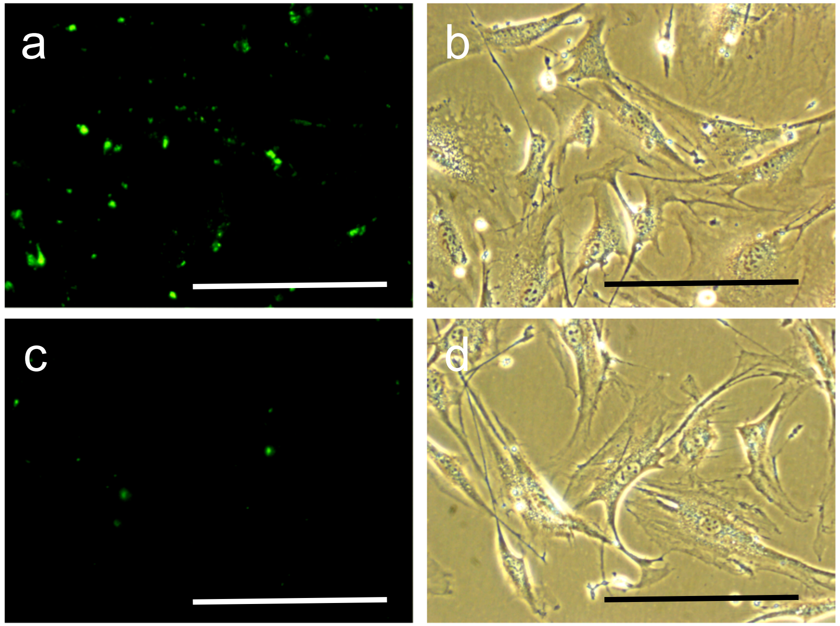

Supplementary Fig. S3

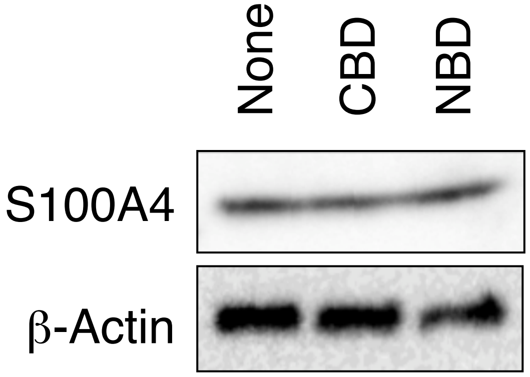

Supplementary Fig. S4

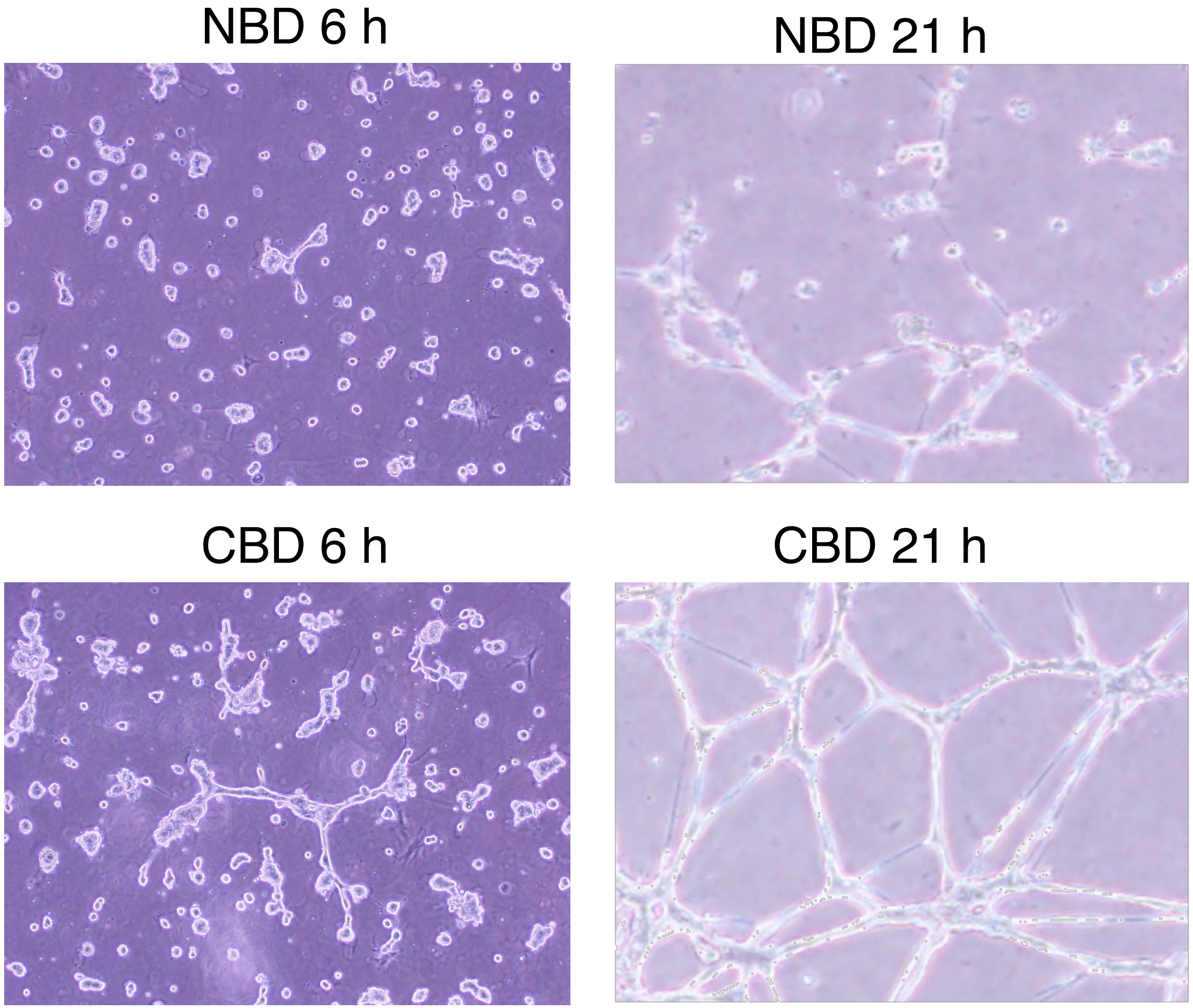

Supplementary Fig. S5

Caspase3/7 activation

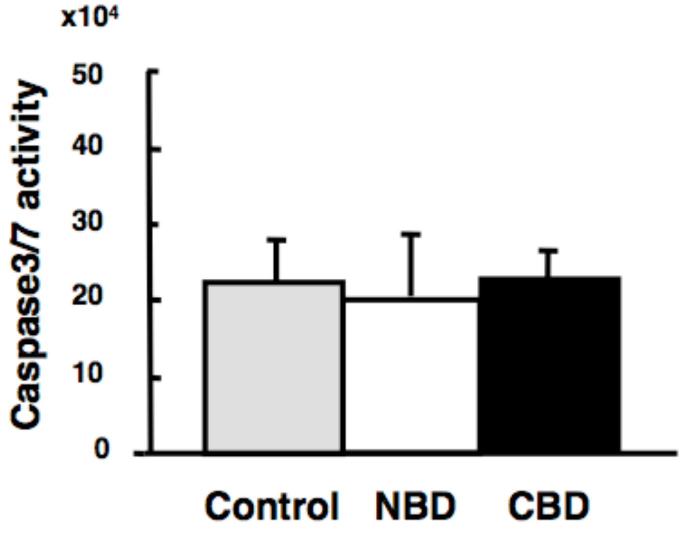

Trypan blue dye exclusion

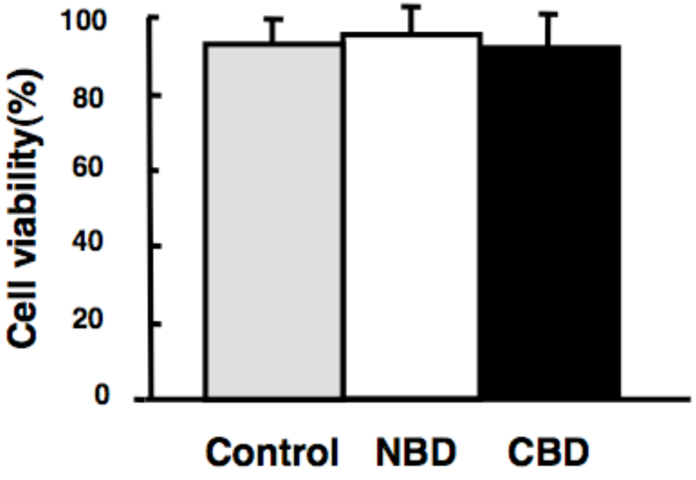

Supplementary Fig. S6

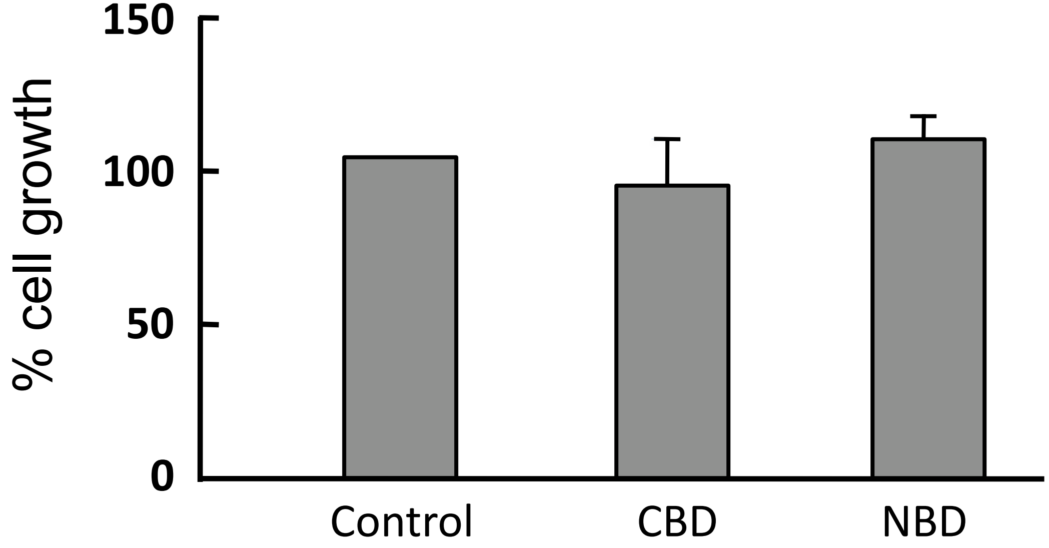

Supplementary Fig. S7

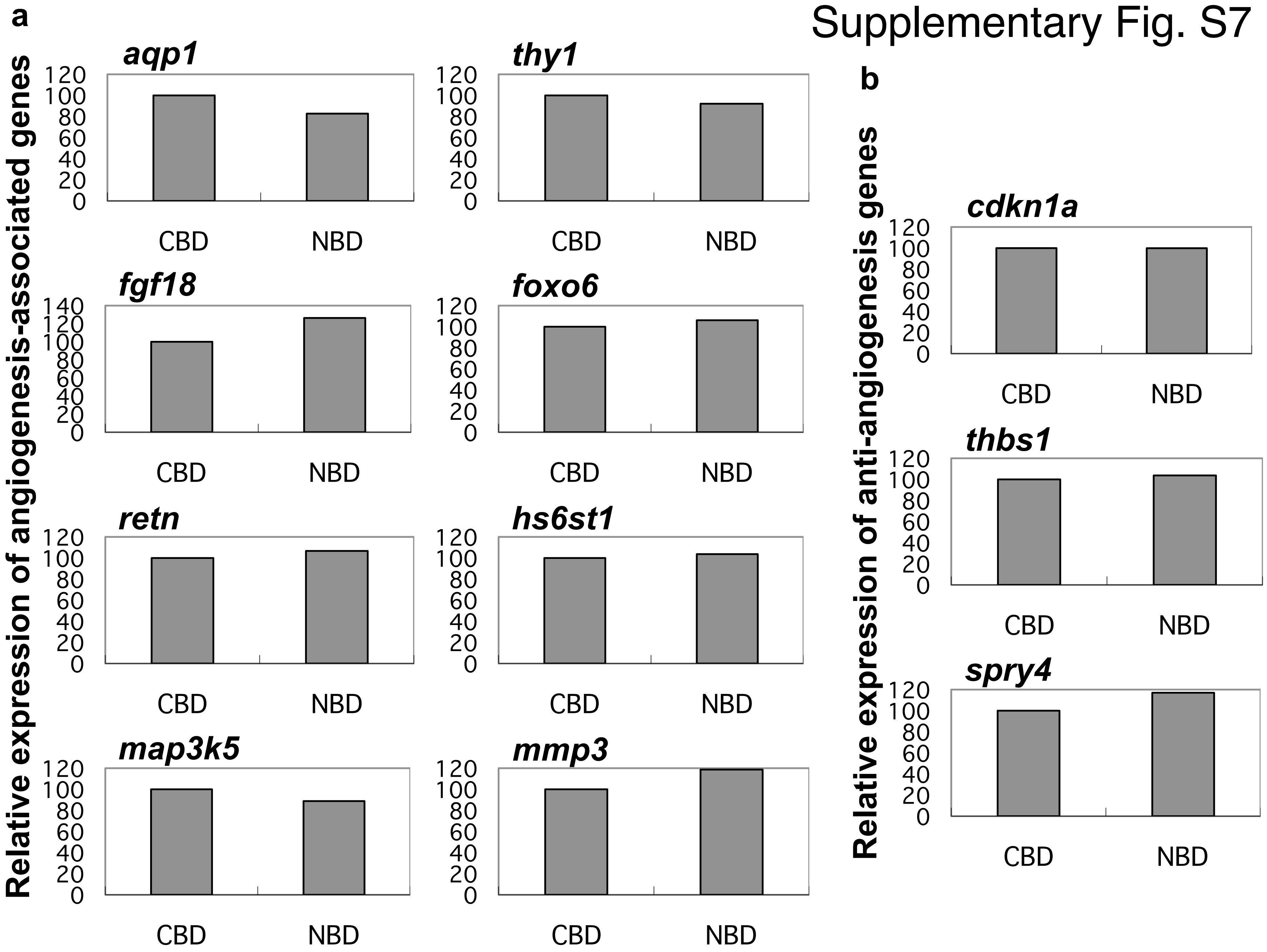

Supplement: Supplementary Figures [file mtm20158-s1.pdf]
